# Deciphering the interplay of gut microbiota and metabolomics in retinal vein occlusion

**DOI:** 10.1128/spectrum.00052-24

**Published:** 2024-07-09

**Authors:** Jing Ai, Yunbo Cao, Cong Zhang, Jun-Hui Sun, Feng Dong, Li Jing, Jianyong Wang, Hongguang Cui

**Affiliations:** 1 Department of Ophthalmology, The First Affiliated Hospital, Zhejiang University School of Medicine, Hangzhou, Zhejiang Province, China; 2 Hepatobiliary and Pancreatic Interventional Treatment Center, Division of Hepatobiliary and Pancreatic Surgery, The First Affiliated Hospital, Zhejiang University School of Medicine, Hangzhou, Zhejiang Province, China; 3 Institute of Translational Medicine, Zhejiang University School of Medicine, Hangzhou, Zhejiang Province, China; Shenzhen Bay Laboratory, Guangming District, Shenzhen, China

**Keywords:** retinal vein occlusion, gut microbiota, 16S rRNA sequencing, metabolomics

## Abstract

This study aims to explore the link between retinal vein occlusion (RVO), a blinding ocular condition, and alterations in gut microbiota composition, to offer insights into the pathogenesis of RVO. Fecal samples from 25 RVO patients and 11 non-RVO individuals were analyzed using 16S rRNA sequencing and liquid chromatography-mass spectrometry (LC-MS). Significant differences in the abundance of gut microbial species were noted between RVO and non-RVO groups. At the phylum level, the RVO group showed an elevation in the ratio of Firmicutes to Bacteroidetes. At the genus level, the RVO group showed higher abundance in *Escherichia_Shigella* (*P* < 0.05) and less abundance in *Parabacteroides* (*P* < 0.01) than the non-RVO group. Functional predictions indicated reduced folate synthesis, biotin metabolism, and oxidative phosphorylation, with an increase in butyric acid metabolism in the RVO group. LC-MS analysis showed significant differences in purine metabolism, ABC transporters, and naphthalene degradation pathways, especially purine metabolism. Pearson correlation analysis revealed significant associations between bacterial genera and fecal metabolites. Enrichment analysis highlighted connections between specific metabolites and bacterial genera. The findings showed that the dysregulation of gut microbiota was observed in RVO patients, suggesting the gut microbiota as a potential therapeutic target. Modulating the gut microbiota could be a novel strategy for managing RVO and improving patient outcomes. Furthermore, the study findings suggest the involvement of gut microbial dysbiosis in RVO development, underscoring the significance of understanding its pathogenesis for effective treatment development.

**IMPORTANCE:**

Retinal vein occlusion (RVO) is a blinding ocular condition, and understanding its pathogenesis is crucial for developing effective treatments. This study demonstrates significant differences in gut microbiota composition between RVO patients and non-RVO individuals, implicating the involvement of gut microbial dysbiosis in RVO development. Functional predictions and metabolic profiling provide insights into the underlying mechanisms, highlighting potential pathways for therapeutic intervention. These findings suggest that modulating the gut microbiota might be a promising strategy for managing RVO and improving patient outcomes.

## INTRODUCTION

Retinal vein occlusion (RVO) is a prevalent blinding disorder within the realm of ophthalmology, ranking as the second most common retinal vascular disease globally (1, 2). Annually, approximately 16.4 million individuals develop RVO (3). The etiology of RVO is multifaceted, with specific pathogenic mechanisms and causative factors yet to be fully elucidated. Associated risk factors for RVO encompass advancing age, diabetes mellitus, hypertension, arteriosclerosis, cardiovascular diseases (characterized by augmented blood viscosity), obesity (accompanied by hyperlipidemia), hematological disorders (leukemia, lymphoma, and polycythemia), dysproteinemias, systemic vasculitis or autoimmune diseases (causing endothelial injury), malignancies, tobacco use, and anemia, among others (4–8). RVO primarily manifests through either partial or complete occlusion of the retinal veins, attributed to these diverse factors. This blockage disrupts venous blood return, leading to venous dilation and subsequent retinal hemorrhage. These initial pathological changes frequently progress to macular edema, which significantly deteriorates visual acuity and can lead to blindness in severe cases (9). Although the incidence of RVO is slightly lower than that of the most prevalent diabetic retinopathy (DR), its prevalence increases gradually with progressive aging, imposing significant economic burdens on both seniors and society (10). Consequently, investigating the pathogenesis underlying RVO holds vital societal and economic values.

Our bodies harbor many ever-evolving microbial communities, known as microbiotas, whose intricate configuration and functionality play crucial roles in our well-being and the onset of diseases (11). The harmonious coexistence of microbial commensals exerts an extensive influence over the intricate web of immune development and homeostasis, shaping the delicate equilibrium between well-being and illness. A growing body of evidence supports the hypothesis that these gut-dwelling allies not only play a role in intestinal disorders but also extend their impact to remote tissues. Emerging scientific evidence has uncovered an intriguing phenomenon termed the “gut–eye axis,” demonstrating how the dynamic presence of gut microbiota influences ocular health, including the retinal structure (12–14).

Various retinal disorders, such as age-related macular degeneration, DR, or retinitis pigmentosa, may fall under the influence of the gut microbiota, hinting at the existence of a direct gut–retina axis (15, 16). However, current research does not directly link RVO onset with gut microbiota imbalance; in other words, whether RVO is related to the gut–retinal axis is still unknown. Systemic diseases that alter blood flow, blood viscosity, coagulation, or vascular structure are known to lead to RVO (17–21), yet the specific pathogenesis remains poorly understood. Therefore, we speculate whether gut dysbiosis could prompt vascular alterations and affect vascular health (i.e., changes in blood viscosity and abnormal blood coagulation), thereby initiating the onset of RVO.

Gut microbes profoundly influence the physiology of their hosts, encompassing vital aspects such as metabolism, immune system homeostasis, nervous system regulation, and resistance against pathogen colonization (22, 23). With the vigorous advancement of multiomics technologies, there has been a shift from meticulous reductionist investigations toward holistic systemic research. These technologies allow for the cross-validation of findings, enhancing the robustness and applicability of our conclusions about gene functionality. However, genomic analysis of gut microbiomes merely deciphers microbial composition and potential functionalities, without delving into their actual activities at a viable state. In contrast, metabolomics represents a discipline that explores the variations in metabolites of a community of organisms resulting from external perturbations and unveils patterns of temporal changes—a field more closely aligned with phenotypic studies (24). Thus, our study endeavors to unravel potential mechanisms underlying the onset of RVO by integrating various omics technologies, including genomics and metabolomics, offering significant implications for guiding clinical therapeutic development.

## RESULTS

### Clinical and demographic characteristics of the study participants

There are no statistical differences between the RVO group and the non-RVO (NRVO) group in demographic and clinical characteristics including age, gender, and BMI (*P* > 0.05) (Table 1).

**TABLE 1:** Baseline characteristics of study participants^*a*^

Characteristic	All individuals (*n* = 36)	NRVO (*n* = 11)	RVO (*n* = 25)	*P-*value
Age (years) [mean (SD)]	60.2 (9.4)	64.5 (6.7)	58.4 (9.9)	0.070
Age (year, %)				0.72
<45	1 (2.8)	0 (0.0)	1 (4.0)	
45–69	30 (83.3)	9 (81.8)	21 (84.0)	
≥70	5 (13.9)	2 (18.2)	3 (12.0)	
Sex (%)				0.27
Female	13 (36.1)	2 (18.2)	11 (44.0)	
Male	23 (63.9)	9 (81.8)	14 (56.0)	
BMI (kg/m^2^) [mean (SD)]	24.1 (2.6)	23.4 (3.0)	24.4 (2.5)	0.26
Diabetes mellitus (%)				
No	27 (75.0)	7 (63.6)	20 (80.0)	0.53
Yes	9 (25.0)	4 (36.4)	5 (20.0)	
Hypertension (%)				
No	19 (52.8)	6 (54.5)	13 (52.0)	0.99
Yes	17 (47.2)	5 (45.5)	12 (48.0)	
Cholesterol (mmol/L) (median [IQR])	4.49 [4.04, 5.35]	4.91 [3.86, 5.06]	4.46 [4.21, 5.42]	0.41
Triglyceride (mmol/L) (median [IQR])	1.52 [0.90, 3.02]	1.57 [0.87, 2.42]	1.44 [1.13, 3.12]	0.93

^*a*^ IQR, interquartile range; SD, standard deviation; *P*-value >0.05 indicates that there is no significant difference between the RVO group and the NRVO group.

### Sequencing of the gut microbiota

We subjected all fecal samples to 16S rRNA gene sequencing. Employing the Illumina NovaSeq sequencing platform, we performed data concatenation, filtering, and quality control denoising processes, culminating in the acquisition of 4,382,803 high-quality 16S rRNA sequencing results. Subsequently, through chimera filtering, we obtained 3,840,669 valid data points for species annotation and abundance analysis, with an average sequence length of 417 bp. Following taxonomic identification, we achieved annotations for a total of 3,316 amplicon sequence variants (ASVs). The Venn diagram revealed 1,142 shared ASVs between the two groups (Fig. S1). Furthermore, the cumulative species boxplot exhibited a gradually attenuating slope toward the right (Fig. S2), indicating sufficient sampling and suggesting that the number of species does not significantly increase with the expansion of sample size, thereby enabling us to proceed with data analysis.

### Alpha diversity and beta diversity of the gut microbiota

**Fig 1:**
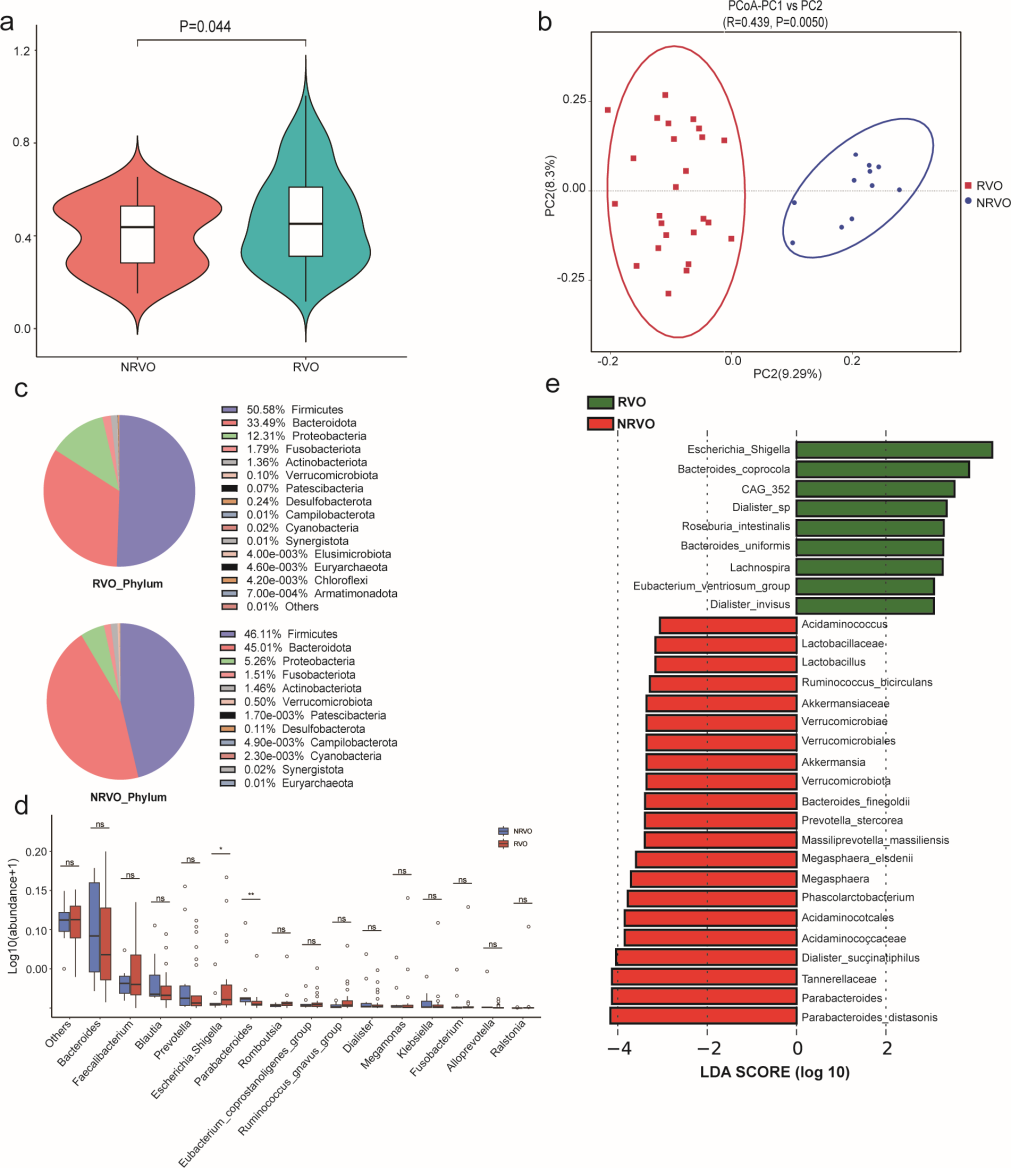
16S rRNA gene sequencing of bacteria in feces between RVO and NRVO groups. (a) Beta diversity analysis based on the weighted UniFrac distance algorithm in two groups. (b) PCoA based on the Jaccard similarity algorithm in two groups. (c) Pie chart illustrating the differential proportion of species at the phylum level in two groups. (d) Bar graph depicting the relative abundance differences at the genus level between two groups (^*^P < 0.05; ^**^P < 0.01; ns, *P* > 0.05). (e) Linear discriminant analysis effect size (LEfSe) analysis of differential species abundances and differential species scores between the RVO and NRVO groups was performed. Green bars indicate relatively high-abundance species in the RVO group, and red bars indicate relatively high-abundance species in the NRVO group.

The Alpha diversity indices, including Chao1, Shannon, Simpson, observed_otus, and Pielou’s evenness, were not statistically different between the two groups (*P* > 0.05, Table 2), indicating a resemblance in microbial richness and evenness. The rank abundance curves also revealed a balanced distribution of species abundance, with no statistical differences observed among the samples (Fig. S3). Beta diversity analysis utilizing the weighted UniFrac distance algorithm, followed by the Wilcoxon rank-sum test, demonstrated significant differences between the RVO and NRVO groups (*P* = 0.044, Fig. 1a). Anosim analysis using the Jaccard algorithm (*R* = 0.44, *P* = 0.0050) indicated that inter-group dissimilarity exceeded intragroup dissimilarity. Furthermore, visualization of the two sample groups using principal coordinate analysis (PCoA) based on Jaccard distances displayed distinct separation between the RVO and NRVO groups (Fig. 1b), suggesting substantial differences in the gut microbiota composition between the RVO and NRVO groups.

**TABLE 2:** Alpha diversity analysis by a Wilcoxon rank-sum test between RVO and NRVO^*a*^

Parameter	NRVO	RVO	*P*-value
Chao1 index (interquartile range [25%, 75%])	385.2, 461.5	409.9, 463.0	0.76
Mean observed_otus index ± SD	420.4 ± 84.2	414.0 ± 94.9	0.84
Pielou’s evenness index (interquartile range [25%, 75%])	0.66, 0.69	0.64, 0.73	0.29
Shannon index (interquartile range [25%, 75%])	5.75, 6.00	5.46, 6.41	0.41
Simpson index (interquartile range [25%, 75%])	0.93, 0.95	0.92, 0.97	0.44

^*a*^ *P*-value >0.05 indicates that there is no significant difference between the RVO group and the NRVO group.

### Taxonomic analysis of the gut microbiome at the phylum and genus levels

At the phylum level, the distribution of microbial abundance between the RVO and NRVO groups is depicted in Fig. 1c. Among these, Firmicutes, Bacteroidota, and Proteobacteria emerge as the three predominant phyla. The ratio between Firmicutes and Bacteroidota in the RVO group shows an increasing trend; however, this difference lacks statistical significance (*P* = 0.10, Wilcoxon rank-sum exact test).

At the genus level, the RVO group demonstrates a significant increase in *Escherichia_Shigella* (*P* < 0.05), while *Parabacteroides* experiences a notable decrease (*P* < 0.01), as illustrated in Fig. 1d. Notably, employing LEfSe analysis (LDA = 3), the RVO group showed higher abundances of *Escherichia_Shigella*, CAG_352, *Lachnospira*, and *Eubacterium_ventriosum*_group at the genus level. At the species level, *Bacteroides_coprocola*, *Dialister*_sp, *Roseburia_intestinalis*, *Bacteroides_uniformis*, and *Dialister_invisus* exhibit higher abundances in the RVO group (Fig. 1e).

### Predicted functional potentials of the altered gut microbiota

**Fig 2:**
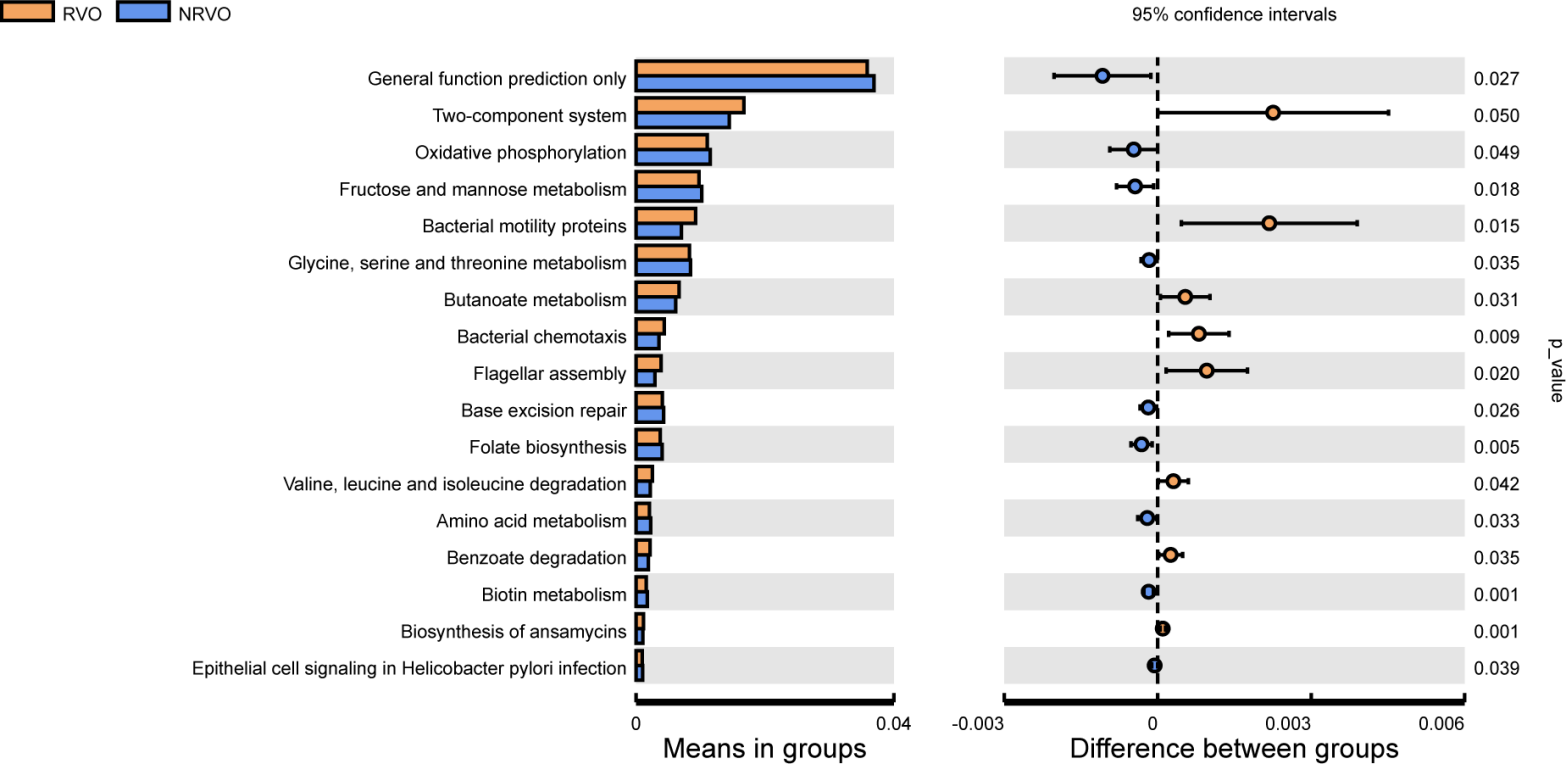
Predicted microbial functional analysis. PICRUSt2 analysis resulting in KEGG Orthology level 3 pathways between RVO and NRVO groups.

To anticipate the potential functionalities arising from gene alterations within the gut microbiota, we utilized Phylogenetic Investigation of Communities by Reconstruction of Unobserved States 2 (PICRUSt2) to explore Kyoto Encyclopedia of Genes and Genomes (KEGG) Orthology level 3 pathways. The results unveiled noteworthy reductions in various metabolic processes including folate biosynthesis; biotin metabolism; oxidative phosphorylation; fructose and mannose metabolism; glycine, serine, and threonine metabolism; and amino acid metabolism, among the RVO group. Conversely, significant increases emerged in butanoate metabolism; valine, leucine, and isoleucine degradation; and benzoate degradation pathways (Fig. 2).

### Untargeted metabolomics analysis of fecal metabolic profiles

**Fig 3:**
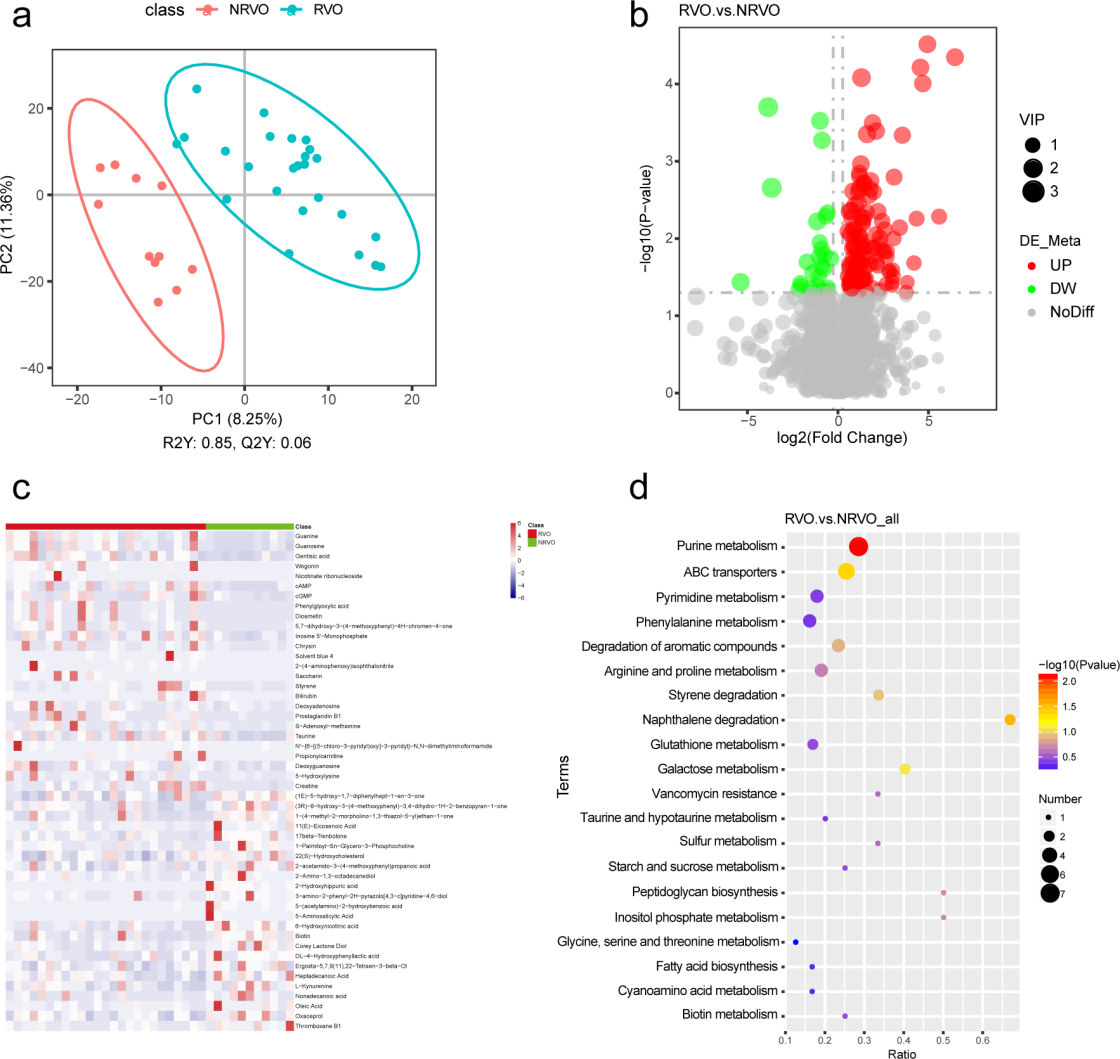
Liquid chromatography-mass spectrometry analysis of fecal metabolite. (a) PLS-DA conducted to compare the NRVO and RVO groups. (b) Volcanic map of differential metabolites. (c) Heat map showing the abundances of the top 50 differential metabolites. (d) Bubble chart of the top 20 enriched KEGG metabolic pathways between RVO and NRVO groups.

By employing liquid chromatography-mass spectrometry (LC-MS) for untargeted metabolomics analysis of fecal metabolites within the gut, a total of 1,799 metabolites were detected, with 150 differential metabolites identified. Utilizing the partial least squares discriminant analysis (PLS-DA) method, a visual representation in the form of a score plot was obtained, wherein each point signifies a sample, reflecting their similarities or dissimilarities in terms of metabolite composition. Notably, the PLS-DA score plot demonstrated a substantial distance between the NRVO and RVO groups, highlighting pronounced disparities between the two groups (Fig. 3a). Hence, discernible variations were evident in the fecal metabolomics profiles between the RVO and NRVO groups. In the volcano plot of differential metabolites (Fig. 3b), each point represents a metabolite, with red indicating significantly upregulated metabolites and green signifying significantly downregulated ones. It is noteworthy that the number of significantly upregulated differential metabolites surpassed that of significantly downregulated metabolites. A selection of the top 50 differentially expressed metabolites was subjected to heatmap analysis, elucidating significant distinctions between the RVO and NRVO groups. Specifically, guanine, cGMP, guanosine, and cAMP exhibited substantial increases in the RVO group, while biotin showcased a significant decrease (Fig. 3c).

### Anticipating the effects of various metabolites on human metabolic pathways

**Fig 4:**
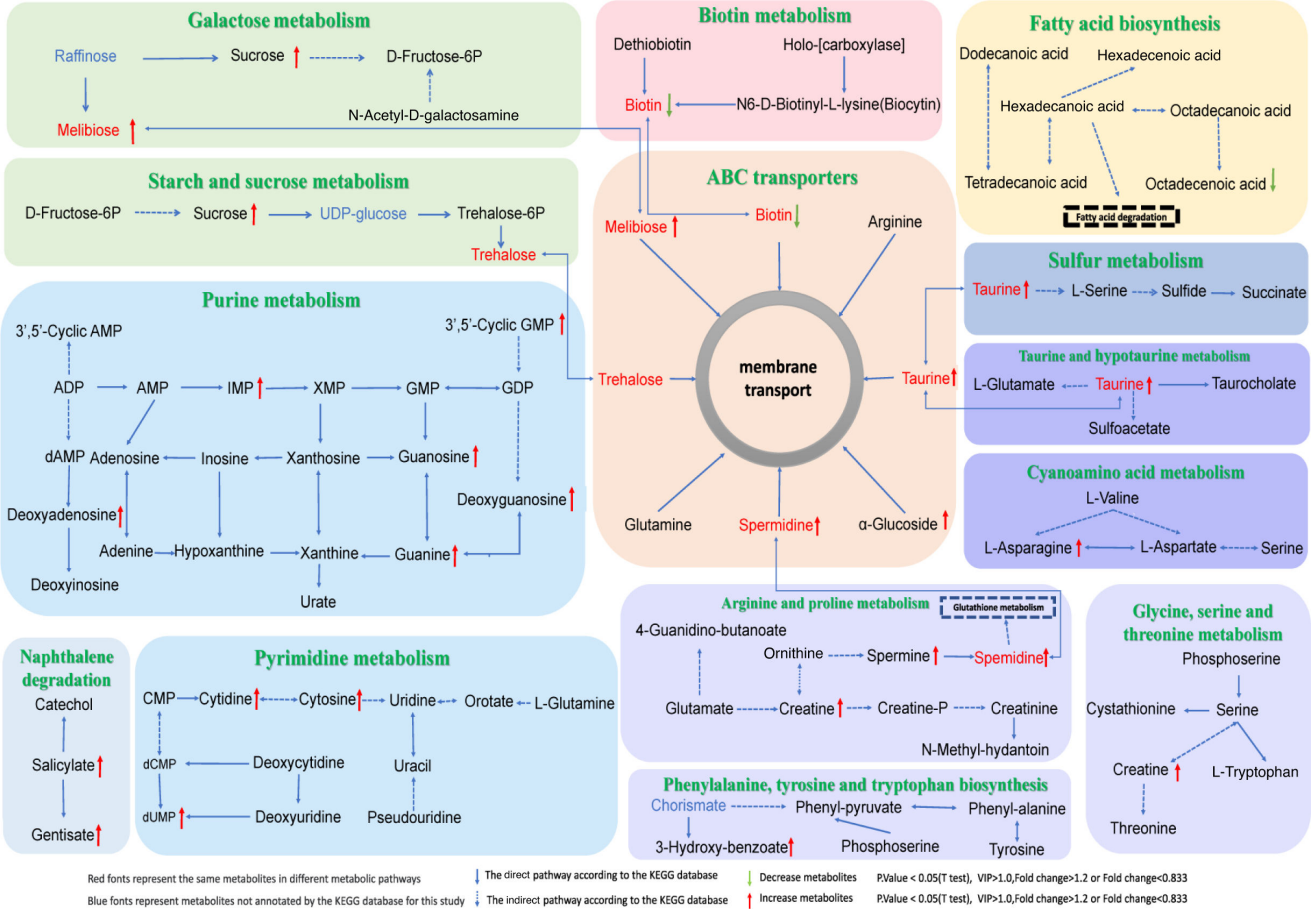
KEGG pathway of the differential metabolites between RVO and NRVO groups (fold change >1.2 or <0.833, VIP ≥ 1.0, *P* < 0.05).

Three distinct metabolic pathways were identified through the KEGG pathway mapper, as indicated in Fig. 3d. These pathways encompass a total of 14 significantly divergent metabolites. Notably, within the purine metabolism pathway, several metabolites exhibited upregulation, including cGMP, deoxyguanosine, inosine 5′-monophosphate, guanosine, guanine, deoxyadenosine, and 3′,5′-cyclic AMP. Conversely, the ABC transporters pathway demonstrated downregulation of biotin while showcasing an elevation in taurine, spermidine, melibiose, and α-glucoside. Furthermore, the naphthalene degradation pathway revealed an upsurge in gentisic acid and salicylic acid. The interplay between these differentially enriched metabolites and their corresponding metabolic pathways in relation to the RVO and NRVO groups is visually depicted in Fig. 4. Additionally, it is noteworthy that these distinctive metabolites are also distributed across 11 other metabolic pathways, encompassing galactose metabolism; biotin metabolism; fatty acid biosynthesis; starch and sucrose metabolism; sulfur metabolism; taurine and hypotaurine metabolism; cyanoamino acid metabolism; pyrimidine metabolism; arginine and proline metabolism; glycine, serine, and threonine metabolism; and phenylalanine, tyrosine, and tryptophan biosynthesis.

### Correlation analysis between 16S rRNA gene sequencing and LC-MS untargeted metabolomics

**Fig 5:**
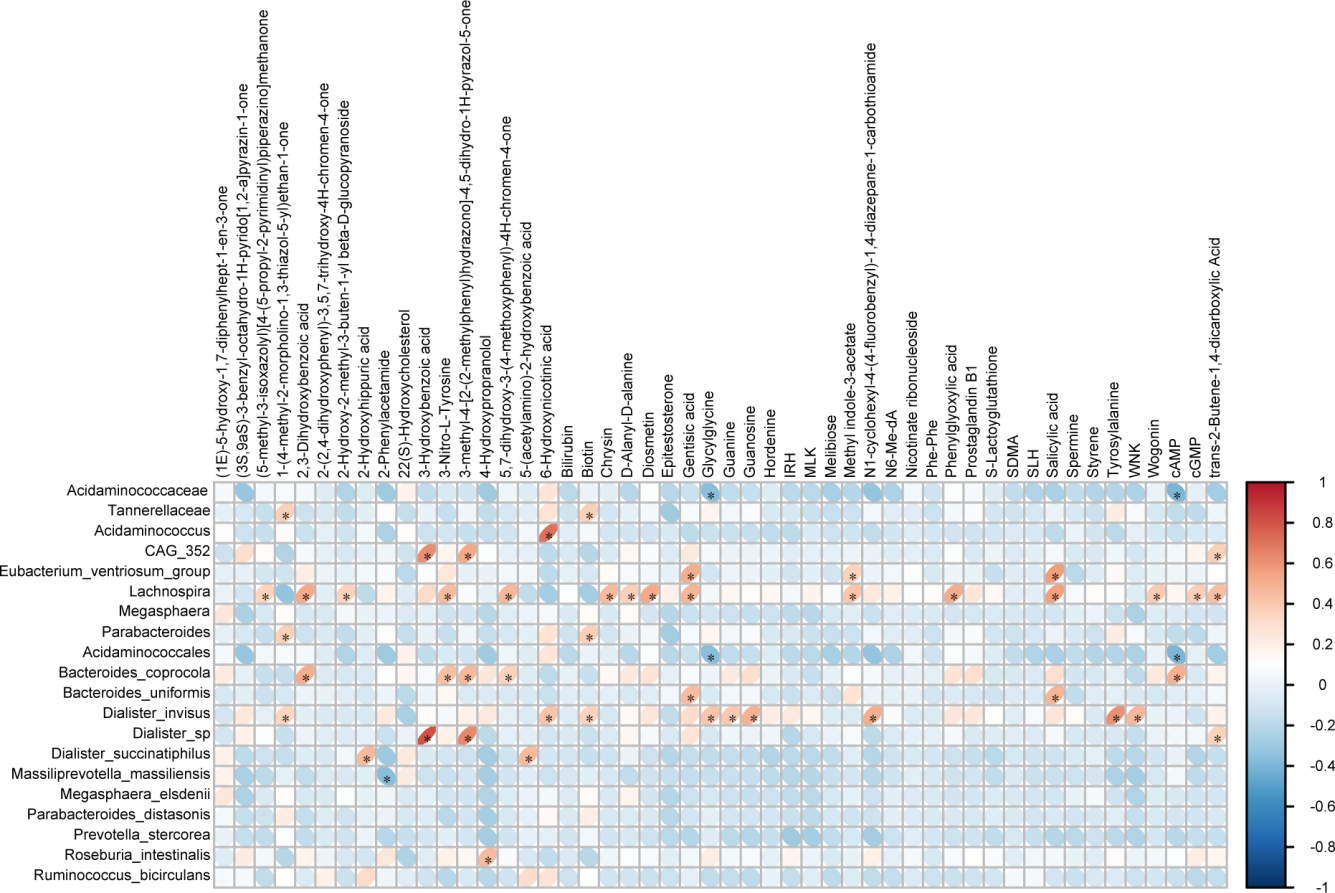
Heat map visualization of the correlation analysis for the top 20 differentially abundant bacterial genera and the top 50 differentially expressed fecal metabolites. In the diagram, the horizontal axis represents differential bacterial genera, while the vertical axis represents differential metabolites. The legend on the right side indicates the correlation coefficient, with red indicating positive correlation and blue indicating negative correlation (^*^P < 0.05).

Using Pearson correlation analysis, we performed an association study between the top 20 significantly different bacterial genera identified through 16S rRNA amplicon sequencing and the top 50 significantly different metabolites obtained from LC-MS metabolomics. To assess the degree of correlation between species diversity and metabolites, a heat map was constructed. We observed a close relationship between 15 distinct bacterial genera and 31 differential fecal metabolites (Fig. 5).

**Fig 6:**
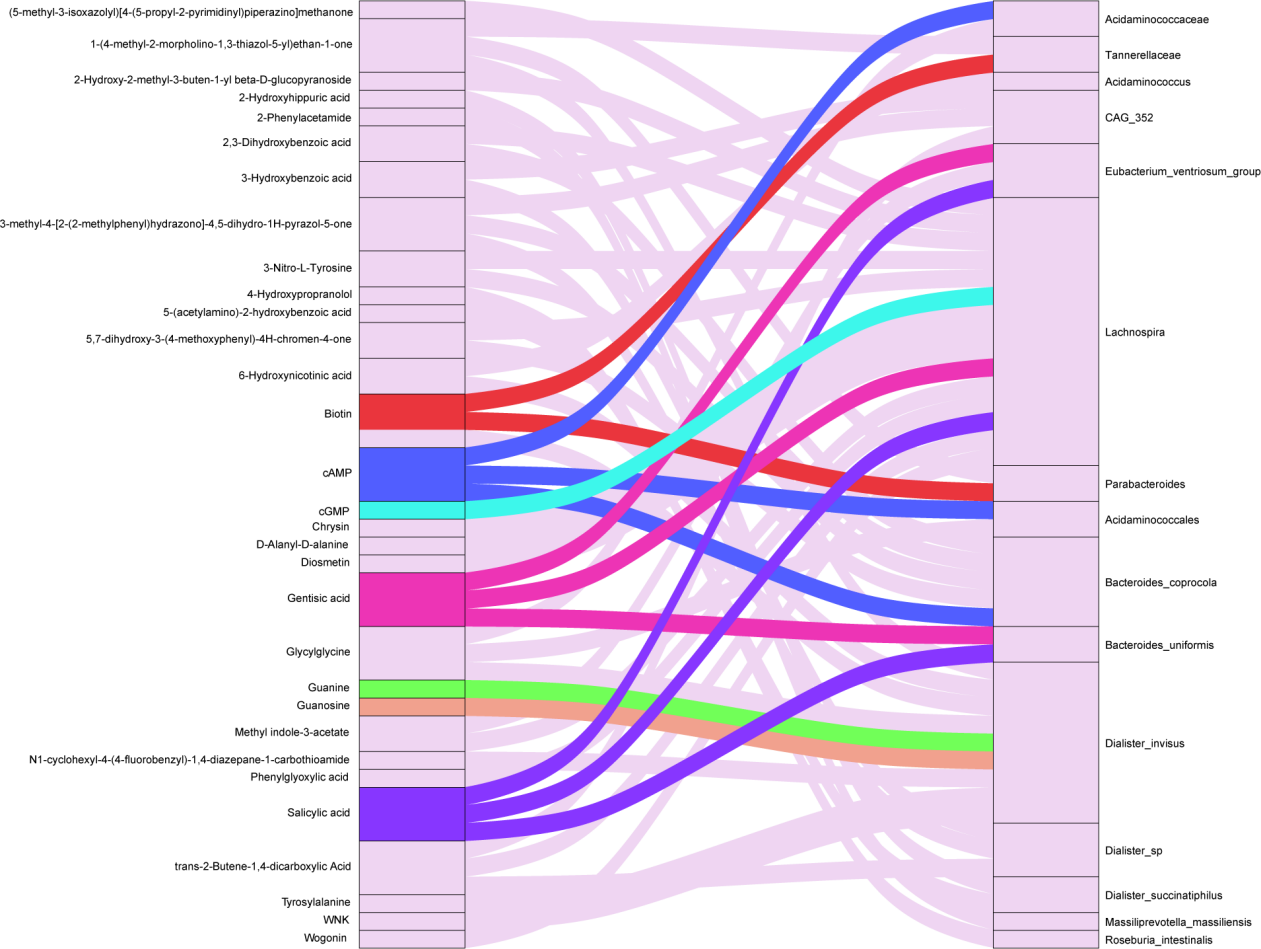
Sankey diagram illustrating the correlations between the differentially expressed fecal metabolites and the differentially abundant bacterial genera. The diagram is constructed based on the correlation results between differential bacterial genera and the top 50 differentially expressed metabolites. A significance threshold of *P* < 0.05 was applied to determine associations. The diagram displays seven differentially expressed metabolites annotated in human KEGG pathways that are correlated with nine differential bacterial taxa (highlighted in color). Additionally, differentially expressed metabolites not annotated in human KEGG pathways are indicated with a background color.

**Fig 7:**
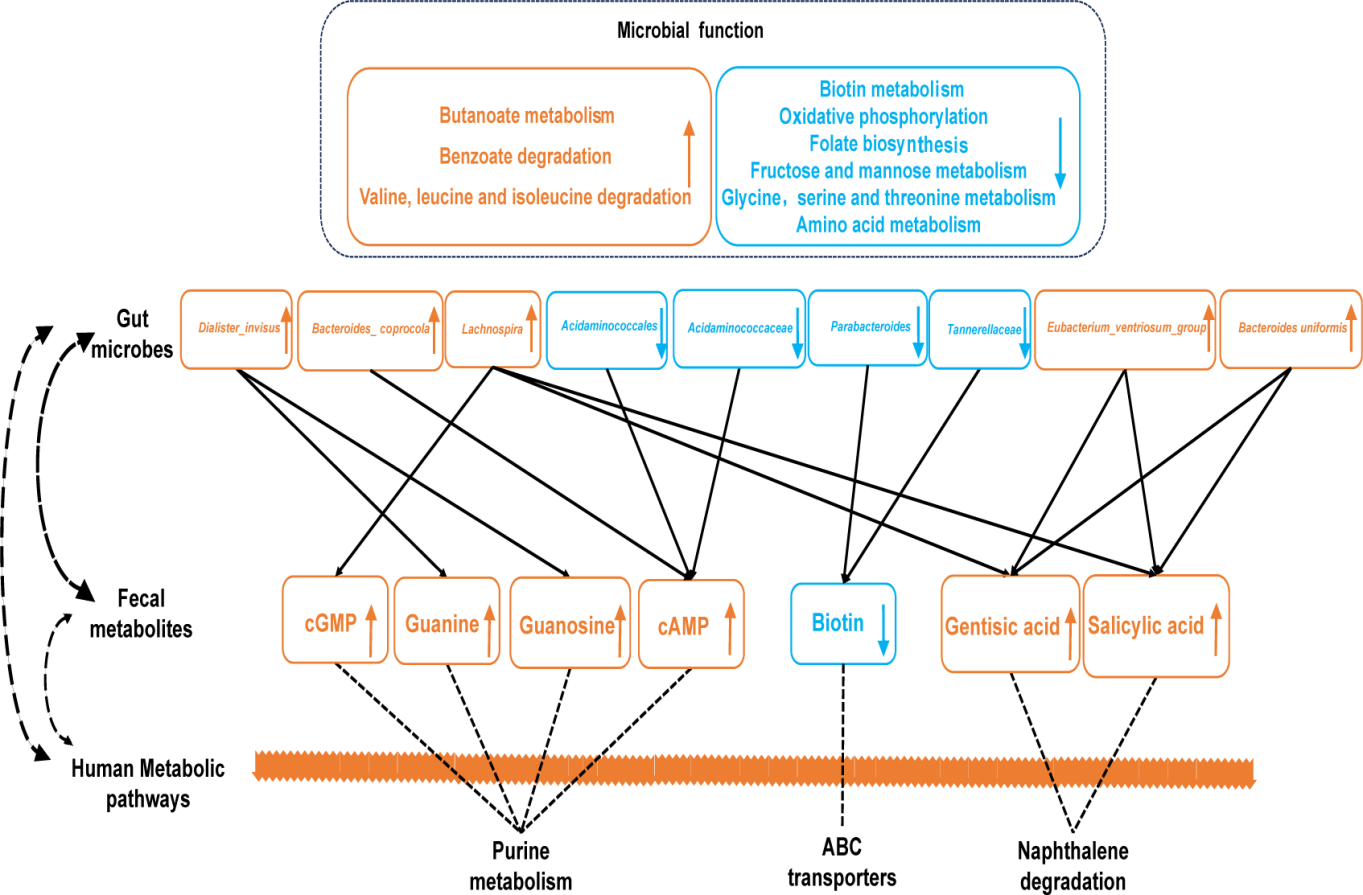
The schematic diagram illustrates the impact of differential gut microbiota on the RVO population. Orange text indicates increased potential pathobionts, fecal metabolites, and microbial functions, while blue text indicates decreased potential probiotics, fecal metabolites, and microbial functions.

Through enrichment analysis of human KEGG pathways, we found a significant association between nine differential bacterial genera and seven differential fecal metabolites (Fig. 6; Table 3). These seven metabolites may have an impact on three specific human metabolic pathways (Table 3). Figure 7 illustrates the relationship between compositional changes in the gut microbiota of the RVO and NRVO groups and differential fecal metabolites as well as metabolic pathways. In the purine metabolism pathway, we observed a significant positive correlation between guanine and the *Dialister_invisus* genus (ρ = 0.41, *P* = 0.01), cGMP and the *Lachnospira* genus (ρ = 0.38, *P* = 0.02), and guanosine and the *Dialister invisus* genus (ρ = 0.50, *P* = 0.002). Furthermore, we observed a significant negative correlation between cAMP and the *Acidaminococcaceae* (ρ = −0.40, *P* = 0.02) and *Acidaminococcales* (ρ = −0.40, *P* = 0.02) genera, as well as a significant positive correlation between cAMP and the *Bacteroides_coprocola* genus (ρ = 0.50, *P* = 0.002). Within the ABC transporters pathway, biotin exhibited a significant positive correlation with the *Tannerellaceae* (ρ = 0.37, *P* = 0.03) and *Parabacteroides* (ρ = 0.37, *P* = 0.03). In the naphthalene degradation pathway, salicylic showed a significant positive correlation with the *Lachnospira* (ρ = 0.56, *P* = 0.0004), *Bacteroides_uniformis* (ρ = 0.46, *P* = 0.005), and *Eubacterium_ventriosum*_group genera (ρ = 0.57, *P* = 0.0003). Gentisic acid showed a significant positive correlation with the *Lachnospira* (ρ = 0.484, *P* = 0.0028), *Bacteroides_uniformis* (ρ = 0.46, *P* = 0.005), and *Eubacterium_ventriosum*_group (ρ = 0.51, *P* = 0.001) (Table 3; Fig. 7).

**TABLE 3:** The correlations between differential bacteria, differential fecal metabolites, and annotated human metabolic pathways^*a*^

Gut microbiota	Microbiota member change	Differential metabolites	Metabolite change	ρ	*P*-value	Human metabolic pathways
*Dialister_invisus*	↑	Guanine	↑	0.41	0.01	Purine metabolism
*Lachnospira*	↑	cGMP	↑	0.38	0.02	
*Dialister_invisus*	↑	Guanosine	↑	0.50	0.002	
*Bacteroides_coprocola*	↑	cAMP	↑	0.50	0.002	
*Acidaminococcales*	↓		↑	−0.40	0.02	
*Acidaminococcaceae*	↓		↑	−0.40	0.02	
*Tannerellaceae*	↓	Biotin	↓	0.37	0.03	ABC transporters
*Parabacteroides*	↓		↓	0.37	0.03	
*Lachnospira*	↑	Salicylic acid	↑	0.56	0.0004	Naphthalene degradation
*Bacteroides_uniformis*	↑		↑	0.46	0.005	
*Eubacterium_ventriosum*_group	↑		↑	0.57	0.0003	
*Lachnospira*	↑	Gentisic acid	↑	0.48	0.003	
*Bacteroides_uniformis*	↑		↑	0.46	0.005	
*Eubacterium_ventriosum*_group	↑		↑	0.51	0.001	

^*a*^ RVO vs NRVO; ↑, upregulation/increasing trend; ↓, downregulation/decreasing trend; ρ, Pearson correlation coefficient between gut microbiota and metabolites. The range of values for the correlation coefficient [−1, 1]. *P*-value <0.05 significant difference in metabolic pathways between the two groups.

## DISCUSSION

The precise etiology underlying the occurrence of RVO remains enigmatic, as any systemic condition that provokes hemodynamic alterations, anomalous blood viscosity, aberrant coagulation propensity, or structural changes within the vascular architecture may instigate the manifestation of this disorder (25). Within the intricate interplay of the gut–retina axis, we hypothesize a possible role of the gut microbiota in modulating vascular dynamics and thereby contributing to the pathogenesis of RVO. In this comprehensive investigation, by employing advanced techniques such as 16S rRNA gene sequencing and LC-MS untargeted metabolomics, we unveiled remarkable discrepancies in the composition of gut microbial communities between individuals afflicted with RVO and those unaffected (NRVO). Notably, an elevated proportion of Firmicutes/Bacteroidetes phyla within the RVO group suggests disruption within the microbial landscape. Furthermore, our analysis reveals 14 distinctive gut-derived metabolites enriched within three pertinent human metabolic pathways, with purine metabolism exhibiting the most striking differences. Pearson correlation analysis reveals close associations between 15 divergent bacterial taxa and 31 distinct fecal metabolites, wherein KEGG pathway enrichment analysis highlighted seven differential metabolites significantly correlated with nine unique microbial genera, thus potentially impacting specific metabolic pathways in humans.

Initially, employing 16S rRNA gene sequencing, we observed a perturbation in the composition of gut microbiota within the RVO group. Notably, beneficial bacteria (12), such as *Lactobacillus*, exhibited reduced abundance. Furthermore, through gene function prediction, we revealed significant reductions in key biological processes, including folate biosynthesis, biotin metabolism, and oxidative phosphorylation, within the RVO group. Conversely, butyric acid metabolism displayed a marked increase.

Folate and biotin are both water-soluble B vitamins. Folate, also known as vitamin B9, plays a critical role in DNA synthesis and maintenance (26), exerting significant effects on the metabolic processes and growth development in organisms. These vitamins can be obtained not only through absorption from food in the small intestine but also from the synthesis by intestinal bacteria, such as *Parabacteroides* and Lactobacillaceae (27). Nonetheless, our analysis using LefSe revealed a notable reduction in the abundance of *Parabacteroides* and Lactobacillaceae bacteria in the RVO group, leading to a corresponding decrease in folate synthesis, consistent with the predicted gene function. Biotin, also known as vitamin B7, is another member of the B vitamins. It participates in cellular metabolic pathways by activating the cGMP/PKG signaling pathway, inducing insulin secretion, and exhibiting immunological and inflammatory functions (28–30). Biotin can be synthesized by bacteria such as *Parabacteroides* and *Lactobacillus* in the gut. Additionally, it serves as a nutritional supplement and immunomodulator to regulate the gut microbiota (31). Our LefSe analysis demonstrated a significant decrease in the bacterial abundance of *Parabacteroides* and *Lactobacillus* in the RVO group, resulting in a corresponding reduction in biotin synthesis, consistent with the predicted gene function. Hyperhomocysteinemia has been identified as a significant risk factor for RVO. Studies have shown that hyperhomocysteinemia promotes cardiovascular diseases (32, 33). It is influenced by B vitamins, particularly folate. Supplementation of folate and other B vitamins effectively lowers plasma total homocysteine levels and improves endothelial function. Therefore, it is plausible that changes in the gut microbiota of RVO patients may lead to reduced synthesis of folate and biotin, thereby affecting vascular endothelial function and promoting the development of RVO.

Oxidative phosphorylation is pivotal for ATP generation through the catabolism of various organic substrates including sugars, fats, and amino acids. This process is tightly regulated and susceptible to disturbances by oxidative stress, a state characterized by an imbalance between pro-oxidants and antioxidants within cellular environments. Importantly, oxidative stress perturbs oxidative phosphorylation, while ATP itself is crucial for moderating oxidative stress effects. In the context of RVO, vascular oxidative stress plays a critical role by promoting vascular inflammation, thrombosis, and alterations in blood properties that are essential for maintaining retinal microcirculation (34–36). The central mechanism involves reactive oxygen species, which induce the peroxidation of polyunsaturated fatty acids in red blood cell (RBC) membranes. This leads to the formation of malondialdehyde, increasing membrane rigidity and, consequently, RBC viscosity. Such changes are particularly problematic in the narrow retinal vessels, increasing the risk of RVO (37). Clinical data support this mechanism, demonstrating higher levels of oxidative stress markers in RVO patients compared to healthy controls, thus highlighting the predictive value of these markers for RVO (32). Additionally, research has identified the role of *Lactobacillus fermentum* in enhancing oxidative phosphorylation in adipose tissues, thereby increasing energy expenditure and preventing diet-induced obesity. Notably, the oral administration of fermented *Lactobacillus* LM1016 has been shown to improve glucose metabolism, alleviate fatty liver in mice fed a high-fat diet, and reduce inflammation in gonadal white adipose tissue (38). In this study, the LefSe analysis shows a reduction in the abundance of *Lactobacillus* in the RVO group, and our predictions of gene function indicated a concurrent reduction in oxidative phosphorylation. These findings suggest a positive relationship between the presence of *Lactobacillus* and the level of oxidative phosphorylation, supporting the hypothesis that a decrease in *Lactobacillus* could lead to reduced oxidative phosphorylation, mitochondrial imbalance, and an enhanced oxidative stress response, thereby disrupting the balance between oxidation and antioxidant processes and potentially contributing to the development of RVO.

Butanoate metabolism encompasses the intricate process within organisms whereby butanoate molecules are transformed and utilized. Butanoate, a short-chain fatty acid, undergoes further oxidation in living organisms to generate energy molecules like pyruvate and contributes to the synthesis of vital physiologically active compounds such as cholesterol and steroid hormones. In certain pathological states, such as diabetes, butanoate metabolism is disrupted (39), potentially leading to hemostatic abnormalities that further exacerbate the onset of RVO (40, 41).

Subsequently, employing untargeted metabolomics analysis using LC-MS, this study successfully identified discernible disparities in purine metabolism within the human metabolic pathways of RVO patients. Metabolites associated with purine metabolism, including cGMP, deoxyguanosine, inosine 5′-monophosphate, guanosine, guanine, and 3′,5′-cyclic AMP, were significantly upregulated. These purine metabolites play pivotal roles in cellular signal transduction and regulate a multitude of physiological and pathological processes, such as inflammation, ischemia, hyperglycemia, hyperlipidemia, and obesity (42, 43). Purine metabolism is part of nucleotide metabolism, encompassing the biosynthesis and degradation of purine derivatives like adenine and guanine within living organisms (44). Higher levels of lactate and purine metabolites in fresh venous blood are deemed potential indicators for early deep venous thrombosis formation (45). RVO denotes a condition characterized by the formation of blood clots in retinal veins. Concurrently, disruptions in purine synthesis and degradation processes, resulting from metabolic disturbances, contribute to increased uric acid synthesis or decreased excretion, leading to hyperuricemia, which in turn triggers vascular disorders such as hypertension and hyperlipidemia (46–48). Hyperlipidemia also represents a significant risk factor for RVO development (32, 49). Thus, it is plausible that perturbations in purine metabolism among RVO patients directly correlate with the pathogenesis of RVO.

To acquire further insights into the intricate interplay between the microbiome and its host, we conducted an in-depth investigation using Pearson correlation analysis to explore the associations between differential microbial taxa and differential fecal metabolites. 16S rRNA sequencing has emerged as a pivotal methodology for elucidating the compositional structure of microbial communities in environmental samples. Metabolomics, on the other hand, enables the measurement of metabolic alterations within the host ecosystem at a specific time point. Thus, to decipher the potential phenotypic changes that may arise from variations in the structure of the host’s microbial community, it becomes imperative to perform a correlation analysis of metabolomics data with microbiota information. Our findings highlight a strong relationship between 15 divergent bacterial genera and 31 dissimilar fecal metabolites. Employing KEGG enrichment analysis, we observed significant correlations between seven enriched metabolites and nine distinct bacterial taxa. Among these, biotin, a key player in metabolism, gene expression, and human diseases (50), demonstrated a pronounced positive association with differential bacterial genera such as *Tannerellaceae* and *Parabacteroides*. The reduction of biotin coincided with a decline in the abundance of *Tannerellaceae* and *Parabacteroides*, indicating their potential role as beneficial microbes implicated in preventing RVO pathology. Notably, studies have suggested that *Parabacteroides* confers advantages by diminishing the risk of chronic diseases in subsequent generations of mice (51). Furthermore, the purine metabolism pathway encompasses vital metabolites including cAMP, guanine, cGMP, and guanosine. Correlation analyses revealed significant associations between these metabolites and distinctive gut bacterial taxa. Specifically, cAMP exhibited a significant positive correlation with *Bacteroides coprocola*. Guanine and guanosine both showed a significant positive correlation with *Dialister invisus*. Additionally, cGMP exhibited a marked positive association with *Lachnospira*. Notably, cAMP also exhibited a noteworthy negative correlation with both *Acidaminococcaceae* and *Acidaminococcales*. These findings suggest that *Acidaminococcaceae* and *Acidaminococcales* may potentially represent beneficial bacterial taxa.

This comprehensive study sheds light on the potential mechanisms underlying RVO by investigating alterations in intestinal microbiota and metabolites. Utilizing advanced techniques such as 16S rRNA gene sequencing and LC-MS untargeted metabolomics analysis, we identified significant differences in gut microbial composition and metabolites between RVO patients and NRVO controls. The findings suggest a close association between abnormal levels of purine metabolites, reduced probiotic populations, and decreased biotin and folate levels, potentially contributing to the pathogenesis of RVO. Our study provides insights into the possibility of improving gut microbial composition through probiotic supplementation and vitamin B intake as a preventive strategy for RVO. However, several limitations should be acknowledged. The observational design of the study limits the ability to establish causality, and further experimental and clinical research is needed. Additionally, our sample source was limited to fecal samples, and mechanistic explanations for the observed associations are lacking. Moreover, our study lacks functional experiments to directly link microbiome changes to RVO-related factors and validation in an independent cohort. Although our findings are currently limited to observed associations and do not definitively establish causality or identify specific strains involved, we are committed to further investigating these relationships. Future work will aim to identify specific strains of *Lactobacillus* that might be particularly influential in this context. Future large-scale studies are necessary to validate our findings. Despite these limitations, this study offers valuable insights into the potential mechanisms underlying RVO, suggesting new avenues for preventive and therapeutic strategies, but requires additional investigation for validation and mechanistic understanding.

## MATERIALS AND METHODS

### Patients and study cohort

This study presents a retrospective, observational series study of patients diagnosed with RVO. All participants provided written informed consent for the collection and publication of their clinical data in this study. The case records of consecutive patients in our study, who presented to our department from September 2021 to December 2022, were reviewed. All patients received a thorough ophthalmologic examination, including slit-lamp microscopy, retinoscopy, a dilated funduscopic examination using a 90-diopter lens, and an assessment of best-corrected visual acuity and intraocular pressure. Diagnosis of the RVO was verified based on results of fundus examination, optical coherence tomography images, and fundus fluorescein angiography findings by two certified ophthalmic specialists. The diagnosis standard was according to the guideline (52). All demographic data of patients including age, sex, and medical clinical data were collected and analyzed. Medical history including hypertension and diabetes mellitus was also collected.

The inclusion criteria for the study were as follows: for the RVO group: individuals newly diagnosed with RVO, aged above 18 years, and providing uncontaminated fresh stool samples using uniform aseptic collectors; for the NRVO group: individuals from the routine outpatient eye examination population without RVO and any other retinal disease. The exclusion criteria involved the following: (1) acute illness or fever within the past 2 months; (2) gastrointestinal disease or use of probiotics in the past 2 months; and (3) previous treatment with antibiotics, antisyphilis or antituberculosis medications, or steroids.

### Fecal sample collection

Fresh fecal samples were collected using sterile fecal collection devices. After collection, the samples were promptly stored in a refrigerator at −80°C until further analysis and testing.

### DNA extraction and 16S rRNA gene sequencing

The fecal samples were subjected to whole-genome DNA extraction using the cetyltrimethylammonium bromide (CTAB) method (53). The purity and concentration of extracted DNA were assessed by 1% agarose gel electrophoresis. An appropriate amount of sample DNA was taken and diluted with sterile water to a concentration of 1 ng/µL. The V3-4 hypervariable region of the bacterial 16S rRNA gene was amplified using universal primers 515F (CCTAYGGGRBGCASCAG) and 806R (GGACTACNNGGGTATCTAAT). The PCR products were subjected to 2% agarose gel electrophoresis, and qualified PCR products were purified using magnetic beads. Quantification was performed through enzymatic labeling, and equimolar pooling of PCR products was carried out based on their concentrations. After thorough mixing, the pooled PCR products were again subjected to 2% agarose gel electrophoresis for quality assessment. For PCR amplification, each PCR reaction mixture contained 15 µL of Phusion High-Fidelity PCR Master Mix (New England Biolabs), 0.2 µM primers, and 10 ng of genomic DNA template. A preliminary denaturation step was performed at 98°C for 1 minute, followed by 30 cycles of denaturation at 98°C for 10 seconds, annealing at 50°C for 30 seconds, and extension at 72°C for 30 seconds, with a final extension step at 72°C for 5 minutes.

Library construction was accomplished using the TruSeq DNA PCR-Free Sample Preparation Kit. After library construction, quantification was performed using Qubit and qPCR. Once the libraries passed the quality control criteria, they were sequenced using the NovaSeq 6000PE 250 platform. Species classification levels included species (s_), phylum (p), order (o), family (f_), genus (g), and species (s).

### Bioinformatic analysis of 16S rRNA gene sequencing

The raw data obtained from the sequencing platform were initially processed by splitting them into individual sample data sets based on their barcode and PCR amplification primer sequences. After removing the barcode and primer sequences, the reads of each sample were merged using FLASH to obtain the original tag sequence data. The resulting tags underwent further processing to remove chimera sequences. These tags were compared against a species annotation database to detect and eliminate any chimera sequences, resulting in the generation of effective tags. The effective tags were then processed using the DADA2 module in QIIME2 (Version QIIME2-202006) software for denoising, ultimately producing ASVs. Each ASV was taxonomically annotated using the classify-sklearn algorithm naive Bayes classifier in QIIME2, establishing the phylogenetic relationships among all ASV sequences. To ensure comparability, normalization was performed based on the sample with the lowest data quantity. Alpha diversity analysis and beta diversity analysis were conducted using the normalized data.

Statistical analysis was performed using R software (Version 4.0.3). Wilcoxon rank-sum tests and *t*-tests were employed to compare Chao1 index, Shannon index, Simpson index, Pielou’s evenness index, and observed_otus index to evaluate alpha diversity. A Wilcoxon rank-sum test based on the weighted UniFrac distance algorithm was applied to compare the microbial community composition between the two sample groups. Anosim analysis utilizing the Jaccard algorithm assessed intergroup and intragroup differences. PCoA visualization analysis using the Jaccard distance algorithm evaluated the differences and similarities between the two sample groups. Wilcoxon rank-sum tests were used to compare differences at the genus level in the microbiota composition between the two groups. Key bacterial taxa that significantly influenced the intergroup differences were identified through LEfSe analysis (54). For functional prediction, PICRUSt2 software was employed, and based on the PICRUSt2 results, *t*-tests were used to analyze different pathways. Pathways with a *P*-value <0.05 were selected, and the top 17 different pathways were visualized for functional analysis of predicted microbial communities.

### Metabolomics analysis of fecal samples

All chemicals and solvents used were of analytical grade or HPLC grade. Water, methanol, acetonitrile, and formic acid were purchased from CNW Technologies GmbH (Düsseldorf, Germany). 2-Chloro-L-phenylalanine was obtained from Shanghai HC Biological Technology Co., Ltd. (Shanghai, China). Samples weighing 60 mg were accurately weighed and transferred to 1.5 mL Eppendorf tubes.Quality Control (QC) samples were prepared by mixing all the samples together. Next, 20 µL of internal standard [2-chloro-1-phenylalanine dissolved in methanol (0.3 mg/mL)] was added to each sample. Metabolites from each sample were collected, filtered through a 0.22 µm microfilter, and transferred to LC vials for storage at −80°C before LC-MS analysis. The Dionex Ultimate 3000 RS ultra-high-performance liquid chromatography system equipped with a Q Exactive Quadrupole-Orbitrap mass spectrometer (Thermo Fisher Scientific, Waltham, MA, USA) with an electrospray ionization source in positive and negative ion modes was used for analysis. The Acquity UPLC column (1.7 µm, 2.1 × 100 mm) was employed. A binary gradient elution system was used for chromatographic separation, consisting of solvent A (water containing 0.1% formic acid) and solvent B (acetonitrile containing 0.1% formic acid), and the separation was achieved using the following gradient: 5%–20% B/0–2 minutes, 20%–60% B/2–4 minutes, 60%–100% B/4–11 minutes, 2-minute holding at 100% B, B/13 100% to 5% to 13.5 minutes, and holding at 5% B/13.5–14.5 minutes, with a flow rate of 0.4 mL/min, column temperature of 45°C, all samples stored at 4°C during analysis, an injection volume of 5 µL, mass-to-charge ratio range of 66.7~1,000.5, full MS scan resolution of 70,000, HCD tandem MS scan resolution of 35,000, and collision energy set to 10, 20, and 40 eV, and the working voltage of the mass spectrometer was spray voltage (positive ion mode: 3,000 V; negative ion mode: 2,500 V), with a sheath gas flow rate of 45 arbitrary units, auxiliary gas flow rate of 15 arbitrary units, and capillary temperature of 350°C. During the entire analysis period, QC was injected at regular intervals (every 10 samples) to provide a set of data for assessing repeatability. Statistical analysis was performed using R software (Version 4.0.3). Metabolomics data were processed using the metaX0 metabolomics data processing software. PLS-DA was employed to establish the relationship between metabolite expression levels and sample categories, and seven rounds of cross-validation were performed to analyze intergroup differences in metabolites and obtain the variable importance in the projection (VIP) values for each metabolite. Two-tailed *t*-tests were used to determine the statistical differences (*P*-values) and fold changes (FC) of metabolites between the two groups. The criteria for differential metabolite selection were VIP > 1.0, *P*-value <0.05, and FC ≥ 1.2 or FC ≤ 0.833. A volcano plot was created to visualize the differential metabolites, and a heatmap using the R package was generated for clustering analysis of the top 50 differential metabolites based on fold change. Differential metabolite functions and metabolic pathways were annotated using the KEGG database, and the metabolic pathway bubble plot was visualized using the ggplot2 package in R. When the *P*-value of a metabolic pathway was <0.05, it was considered significantly enriched.

### Statistical analysis

The IBM SPSS Statistics 26 software and R software (Version 4.0.3) were used for statistical analysis. Count data were expressed as percentages (%). For normally distributed continuous variables, the mean ± SD was used to summarize the data. Skewed continuous measures were represented by the median and interquartile range [*M* (P25, P75)]. Two independent samples *t*-test was used to compare normally distributed continuous measures, while the Wilcoxon rank-sum test was used for comparing the skewed continuous measures. The chi-squared test was used for comparing categorical measures between two groups. In R software (Version 4.0.3), Pearson correlation analysis was performed to evaluate the correlation between differentially abundant bacteria and the top 50 differential metabolites in feces. The corrplot package in R was used to create a correlation heatmap. Two-sided *P*-value <0.05 indicates statistical significance.

## Data Availability

The microbiome raw data have been deposited in the NCBI database under accession number PRJNA1052399, and metabolomics raw data have been deposited in the MetaboLights database (www.ebi.ac.uk/metabolights/MTBLS8789). All data that support the ﬁndings of this study are included in the manuscript and the supplemental material.

## SUPPLEMENTAL MATERIAL

Supplemental figures spectrum.00052-24-s0001.docx Fig. S1-S3.

ASM does not own the copyrights to Supplemental Material that may be linked to, or accessed through, an article. The authors have granted ASM a non-exclusive, world-wide license to publish the Supplemental Material files. Please contact the corresponding author directly for reuse.
