# Supplementary material for: Deciphering the interplay of gut microbiota and metabolomics in retinal vein occlusion
Source: Microbiol Spectr. 2024 Jul 9;12(8):e00052-24. doi: 10.1128/spectrum.00052-24 (PMC11302663; doi:10.1128/spectrum.00052-24)
Supplement: Supplemental figures — Fig. S1-S3. [file spectrum.00052-24-s0001.docx]

**Supplementary Information**

**Deciphering the interplay of gut microbiota and metabolomics in retinal vein occlusion**

Jing Ai^1†^, Yunbo Cao^2, 3†^, Cong Zhang^2†^, Jun-Hui Sun^2^, Feng Dong^1^, Li Jing^2^, Jianyong Wang^1*^, Hongguang Cui^1*^

1. Department of Ophthalmology, The First Affiliated Hospital, Zhejiang University School of Medicine, Hangzhou 310003, Zhejiang Province, China

2. Hepatobiliary and Pancreatic Interventional Treatment Center, Division of Hepatobiliary and Pancreatic Surgery, The First Affiliated Hospital, Zhejiang University School of Medicine, Hangzhou 310003, Zhejiang Province, China

3. Institute of Translational Medicine, Zhejiang University School of Medicine, Hangzhou, China

†There author contributes equally

*Co-Corresponding authors, Email: wangjy4@zju.edu.cn

*Corresponding authors, Email: 1189002@zju.edu.cn


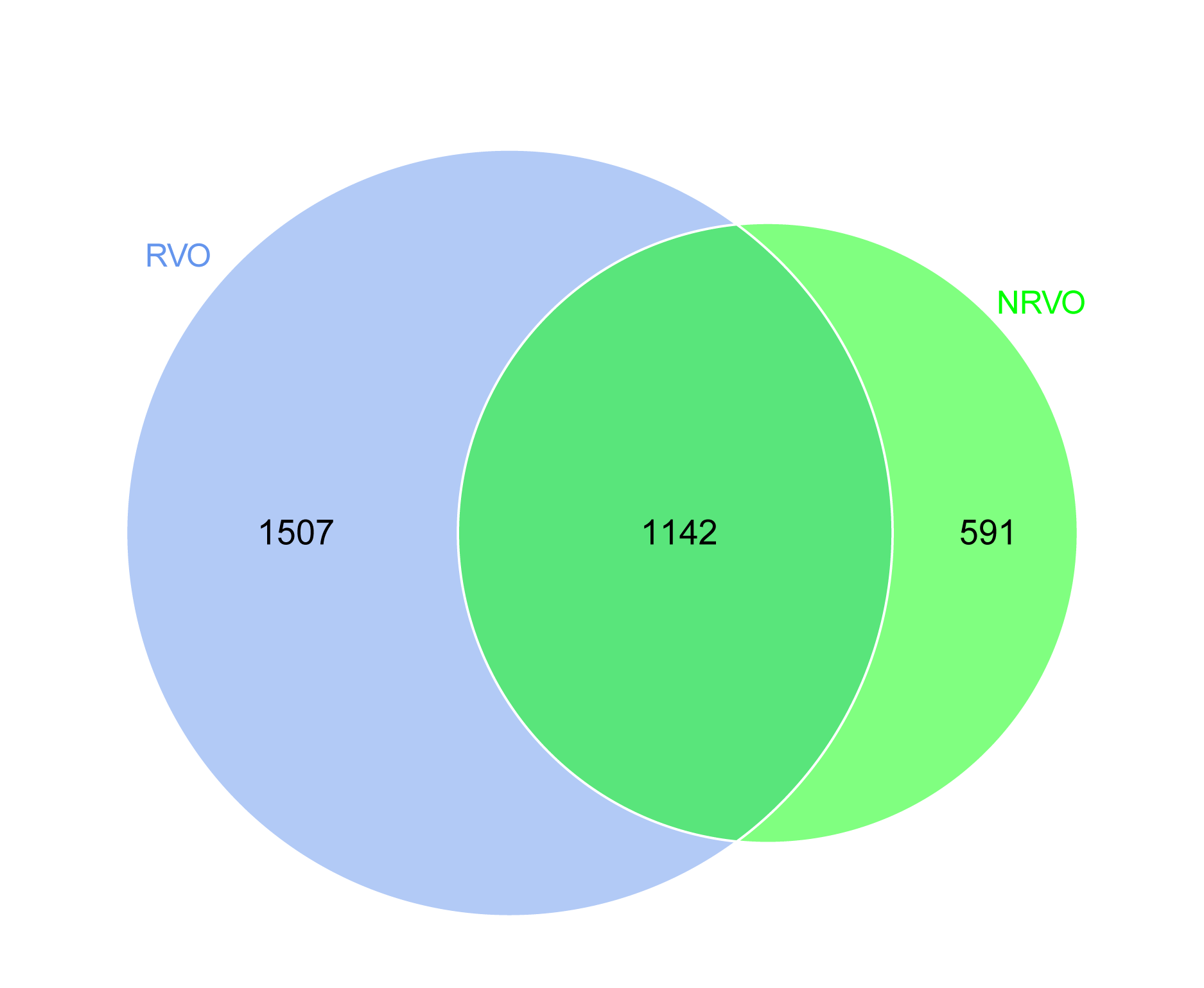


**FIG S1 16S rRNA gene sequencing of bacteria in feces.** Based on the annotated feature sequences, a Venn diagram was constructed to visualize the shared features between the RVO and NRVO groups, which consists of 1142 ASVs.


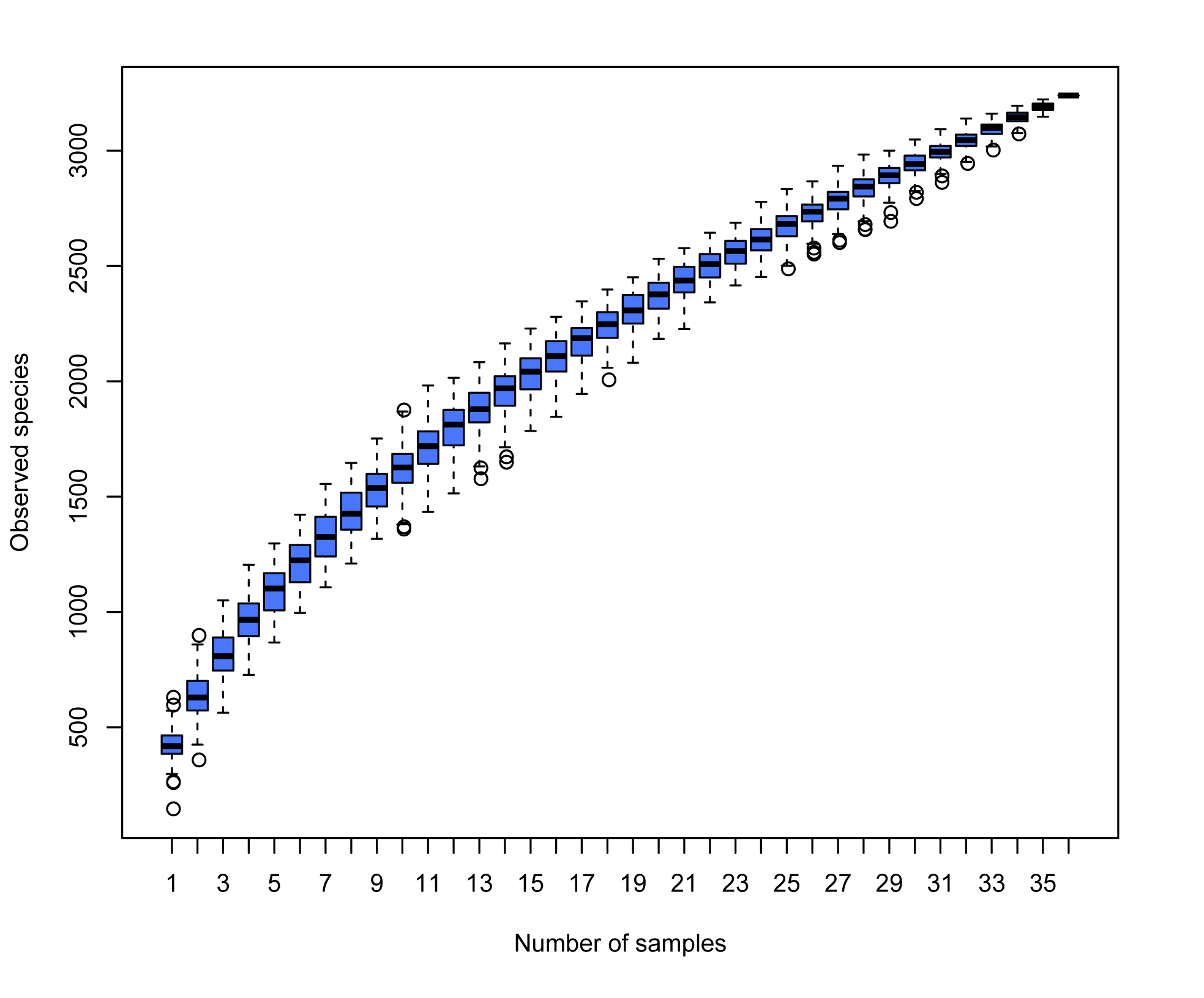


**FIG S2 Species accumulation boxplot.** The x-axis represents the number of samples, while the y-axis represents the cumulative species count (or the number of sampled feature sequences). As the number of samples increases, the cumulative species count levels off, indicating an adequate sample size.


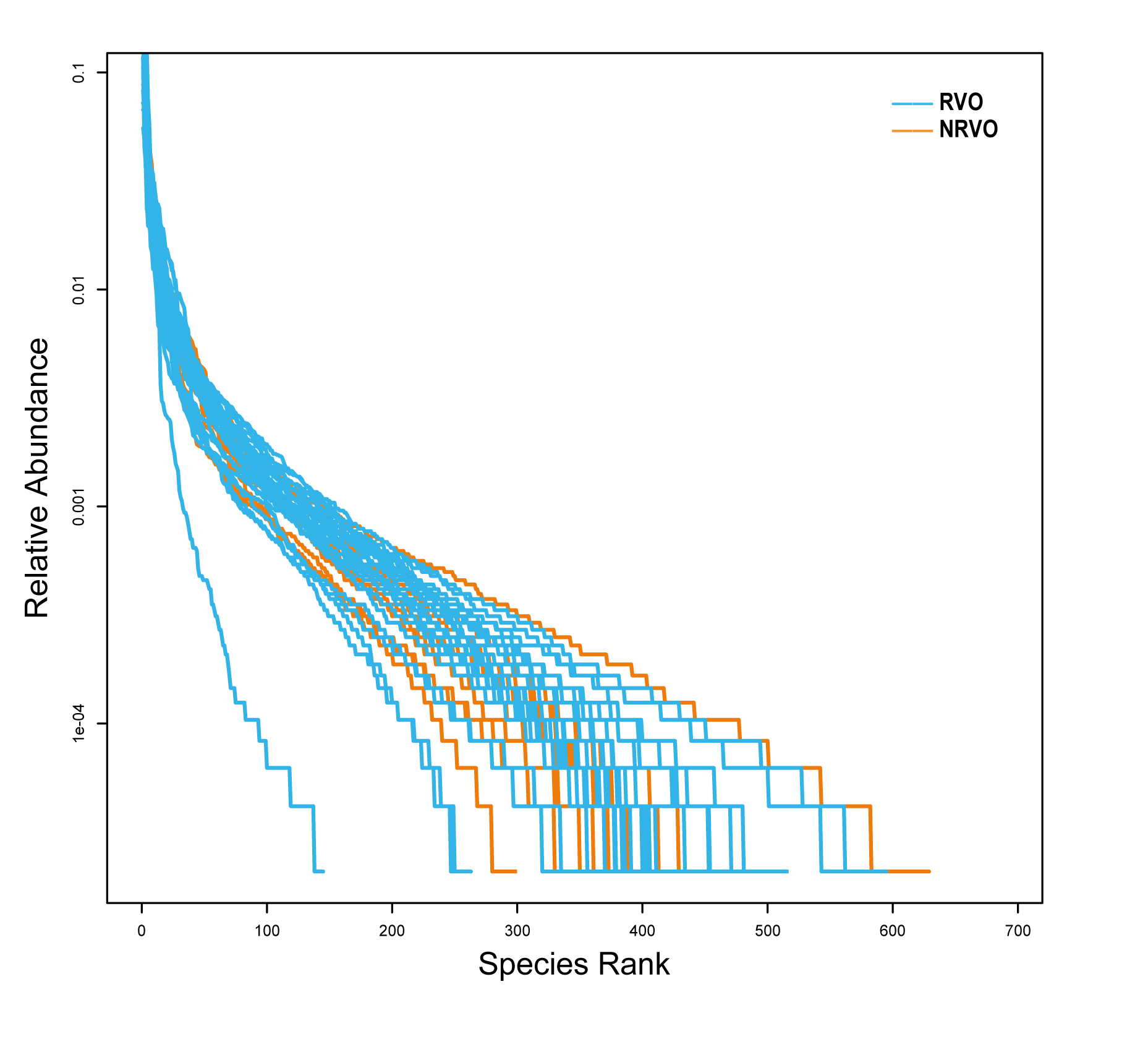


**FIG S3 Rank Abundance curve.** On the horizontal axis, species are sorted according to the number of sequences they contain. The vertical axis represents the relative abundance of the species.
